# Biomarker prediction in autism spectrum disorder using a network-based approach

**DOI:** 10.1186/s12920-023-01439-5

**Published:** 2023-01-23

**Authors:** Maryam Rastegari, Najmeh Salehi, Fatemeh Zare-Mirakabad

**Affiliations:** 1 Department of Mathematics and Computer Science, Amirkabir University of Technology (Tehran, Polytechnic), 424, Hafez Ave, P.O. Box: 15875-4413, Tehran, Iran; 2 School of Biological Science, Institute for Research in Fundamental Sciences (IPM), Tehran, Iran; 3 National Institute of Genetic Engineering and Biotechnology (NIGEB), Tehran, Iran

**Keywords:** miRNA, Co-expression network, Gene expression, Set cover

## Abstract

**Background:**

Autism is a neurodevelopmental disorder that is usually diagnosed in early childhood. Timely diagnosis and early initiation of treatments such as behavioral therapy are important in autistic people. Discovering critical genes and regulators in this disorder can lead to early diagnosis. Since the contribution of miRNAs along their targets can lead us to a better understanding of autism, we propose a framework containing two steps for gene and miRNA discovery.

**Methods:**

The first step, called the FA_gene algorithm, finds a small set of genes involved in autism. This algorithm uses the WGCNA package to construct a co-expression network for control samples and seek modules of genes that are not reproducible in the corresponding co-expression network for autistic samples. Then, the protein–protein interaction network is constructed for genes in the non-reproducible modules and a small set of genes that may have potential roles in autism is selected based on this network. The second step, named the DMN_miRNA algorithm, detects the minimum number of miRNAs related to autism. To do this, DMN_miRNA defines an extended Set Cover algorithm over the mRNA–miRNA network, consisting of the selected genes and corresponding miRNA regulators.

**Results:**

In the first step of the framework, the FA_gene algorithm finds a set of important genes; TP53, TNF, MAPK3, ACTB, TLR7, LCK, RAC2, EEF2, CAT, ZAP70, CD19, RPLP0, CDKN1A, CCL2, CDK4, CCL5, CTSD, CD4, RACK1, CD74; using co-expression and protein–protein interaction networks. In the second step, the DMN_miRNA algorithm extracts critical miRNAs, hsa-mir-155-5p, hsa-mir-17-5p, hsa-mir-181a-5p, hsa-mir-18a-5p, and hsa-mir-92a-1-5p, as signature regulators for autism using important genes and mRNA–miRNA network. The importance of these key genes and miRNAs is confirmed by previous studies and enrichment analysis.

**Conclusion:**

This study suggests FA_gene and DMN_miRNA algorithms for biomarker discovery, which lead us to a list of important players in ASD with potential roles in the nervous system or neurological disorders that can be experimentally investigated as candidates for ASD diagnostic tests.

**Supplementary Information:**

The online version contains supplementary material available at 10.1186/s12920-023-01439-5.

## Background

Autism spectrum disorder (ASD), as a neurodevelopmental disorder, is characterized by limitations in social interaction and communication alongside repetitive, restricted behavioral, interest, or activity patterns [1]. These symptoms are typically diagnosed in children during their second year of life (12–24 months) [1]. It should be noted that it is essential to diagnose these children early and begin behavioral therapy and drug therapy as soon as possible [2]. Therefore, identifying candidate ASD risk factors for diagnosis and finding appropriate drugs for early treatment of this condition can significantly improve the future life quality of autistic children. Although some studies showed the role of immunological, neurological, and environmental factors in this condition, the main reason for ASD is the genetic basis [3, 4]. The phenotype and pathophysiological mechanisms of ASD are highly heterogeneous and complex [5, 6]. However, the characterization of gene expression profiles of ASD cases is a way to detect genetic causes, the effect of non-genetic risk factors on the gene expressions causes an understanding of the pathophysiological mechanisms in ASD [3]. In this regard, some studies focus on finding genes involved in autism with statistical, data mining, and machine learning approaches. For example, Latkowski and Osowski utilized eight different data mining methods independently, each followed by a genetic algorithm to find the most related genes to autism [7]. Another study detected important genes in autism using GBPSO-SVM, a Geometric Binary Particle Swarm Optimization with a Support Vector Machine as a fitness function [8]. They ran a tenfold GBPSO-SVM independently on three sets of genes having a higher rank in three statistical tests. The important genes are proposed based on the frequency of genes in optimal solutions [8]. Kong and his colleagues ranked genes based on differential expression between sample groups. They repeatedly used cross-validation to select some of the top genes from the ranked list. Then, a set of 55 genes is proposed as important genes [9].

MicroRNAs (miRNAs) are a class of non-coding RNAs that play critical roles in regulating gene expression. They target messenger RNAs (mRNAs), the coding RNAs that encode proteins, and activate or repress the translation of mRNAs [10]. So, changes in miRNA expression can affect protein production and cause various problems, including nervous system diseases and disorders [11]. The difference in miRNA expression is also detected in autistic patients compared to normal individuals [12–14]. In this regard, some studies focus on discovering miRNAs involved in autism. Shen and his colleagues used the gene expression profiles of ASD ones to calculate the differentially expressed genes (DEGs) relative to control ones. Then, a sub-network is extracted from an experimentally validated and computationally predicted miRNA-mRNA network by mapping DEGs onto it. Finally, 11 miRNAs are proposed as biomarkers for autism based on three self-defined parameters [15]. Another study constructed a co-expression network for a group of male samples and detected eight dysfunctional modules in autism related functionally to metabolism, immunity, neurodevelopment, and signaling. They also reported miRNAs and transcription factors for each module [16].

As mentioned, some studies try to find a small set of genes involved in autism and some others concentrate on finding relevant miRNAs. Although there is evidence that miRNAs and their corresponding targets might contribute to understanding autism [17], there are few studies that focus on investigating both. Hence, we propose a framework in two steps, one for finding a small set of genes involved in autism and the other for finding a minimum set of miRNAs regulators. In the first step, we offer an algorithm named FA_gene which is based on the gene co-expression network and Protein–Protein Interaction networks. In this way, instead of considering genes individually as in most statistical and machine learning studies, we benefit from a module-based view. In the second step, which is called DMN_miRNA algorithm an mRNA–miRNA is constructed based on the genes obtained from the first step and corresponding miRNA regulators. Then, we use a combinatorial-graph based method by defining a set cover problem over the mRNA–miRNA network. Based on the first step, we find 20 genes as abnormal genes in autism, and the second step announces five miRNAs targeting abnormal genes. Finally, we evaluate the results using previous studies and enrichment analysis on the target genes of the detected miRNAs. Therefore, in this study, a framework consisting of different statistical and systems biology approaches has been proposed to investigate both genes and miRNAs involved in autism.

## Methods

This paper aims to reduce genes involved in autism and find a minimum set of miRNAs (as much as possible) that are effectively related to these genes. In this section, we first declare some notations and describe the extracted gene expression dataset for control and autism samples. Then, we represent a computational framework for miRNA discovery in autism.

## Notations

Two sets, $S = \left\{ {s_{1} , \ldots s_{n} } \right\}$ and $G = \left\{ {g_{1} , \ldots g_{m} } \right\}$, with $n$ individuals and $m$ genes, are defined as sample and gene sets, respectively. The gene expression matrix of gene set $G$ in the sample set $S$ is shown by ${\mathbb{E}}_{n \times m}^{S,G}$ where

$${\mathbb{E}}^{S,G} \left[ {i,j} \right] = {\text{gene expression }}g_{j} {\text{ in individual}} s_{i} .$$

We represent two autistic and control sample sets with $S^{C}$ and $S^{A}$, respectively. Gene expression $g_{j}$ across control and autistic samples is determined by column *j* of the ${\mathbb{E}}^{{S^{C} ,G}}$ and ${\mathbb{E}}^{{S^{A} ,G}}$ matrices, ${\mathbb{E}}^{{S^{C} ,G}} \left[ j \right]$ and ${\mathbb{E}}^{{S^{A} ,G}} \left[ j \right]$, respectively. A set of miRNAs is represented by $R = \left\{ {r_{1} , \ldots r_{l} } \right\}$, where $\left| R \right| = l$ shows the number of miRNAs.

### Extracting control and autism gene expression dataset

Peripheral blood samples of ASDs and control ones (from GSE18123 [9]), which are more accessible than brain tissue samples [18, 19], are retrieved from the Gene Expression Omnibus (GEO) database [20]. Gene expression of these samples is achieved using two GPL570 and GPL6244 platforms with 103 and 182 samples, respectively. We use the GPL6244 platform, which has more samples. Based on the diagnosis, samples are grouped into four categories: CONTROL (81 samples), AUTISM (40 samples), PDD-NOS (47 samples), and ASPERGER’S DISORDER (14 samples). The first and second categories show samples without and with autism, respectively. The third represents the samples that do not fully meet autism criteria (called Pervasive Developmental Disorder-Not Otherwise Specified). The last one indicates samples diagnosed with Asperger disorder (typically have stronger verbal language skills and intellectual ability than autistic samples). This study analyzes samples with CONTROL and AUTISM diagnoses. Besides the diagnosis of autism, there are also some other features for each sample, such as developmental or speech disorder situations and infection to other psychiatric disorders or neurological disorders. We extract samples with no neurological, psychiatric, developmental, or speech disorder to remove the effect of any neurological or psychiatric disorder such as Seizures, Landau-Kleffner Syndrome, Bipolar, or Attention Deficit Hyperactivity Disorder. Though, 77 samples remain for the control group and 21 for autism. We also remove drug-treated controls and those who were allergic to have a purer set of control samples, resulting in 60 control samples. Using all remaining control samples, which are three times larger in size than the autistic sample, biases the variance toward the control samples (in the second step of the first algorithm). Hence, using unequal-sized groups affects the power parameter (fourth and fifth steps of the first algorithm), co-expression network, and changes the connectivity pattern and module extracting. To generate a balanced dataset of control and autistic samples, we select 21 out of 60 control samples. Therefore, we have two sample sets $S^{A}$ and $S^{C}$ with sizes $n_{A} = 21$ and $n_{C} = 21$ for autism and control samples, respectively. Each sample has 32,321 probes in raw files. We use the “Affy” [21] package in R for RMA normalization to construct gene expression matrices from raw files. We also use “annotate” [22] and “hugene10sttranscriptcluster” [23] packages in R to convert probe IDs into gene symbols to achieve a set $G = \left\{ {g_{1} , \ldots g_{m} } \right\}$ with $m = 18801$ gene symbols. Finally, we obtain a gene expression profile for control and autistic samples named ${\mathbb{E}}_{21 \times 18801}^{{S^{C} {,}G}}$ and ${\mathbb{E}}_{21 \times 18801}^{{S^{A} {,}G}}$, respectively.

The details of the selected dataset are available in Table 1. The first and second columns indicate the autism and control samples, respectively. The third and fourth columns display the number of females and males in autism and control samples separately. The last column shows the number of genes for each sample.

**Table 1:** The details of the extracted dataset from GPL6244 for the control and autistic samples. A and C stands for autism and control, respectively

$\left| {S^{A} } \right| = n_{A}$	$|S^{C} | = n_{C}$	Number of females		Number of males		$\left| G \right| = m$
21	21	A	C	A	C	18,801
		4	6	17	15	

We also performed differential gene expression analysis using the “Limma” R package [24] to detect genes with different expressions (DEGs) between control and autism. This package performs t-tests to find up-regulated and down-regulated genes. Here, genes with higher absolute log fold-change of than one and adjusted p-value less than 0.05 were selected as DEGs between autism and control.

### Proposed framework for predicting involved miRNAs in autism

This paper presents a new framework including two main steps for predicting a minimum set of involved miRNAs in autism as follows:

1. Introducing the FA_gene algorithm to find a small set of genes, $G^{A} \subseteq G$, involved in autism as an abnormal gene set.
2. Introducing the DMN_miRNA algorithm to detect the minimum number of miRNAs, $\mathop R\limits^{\prime } \subseteq R$, as regulators covering (targeting) the gene set $G^{A}$.

Each step of our framework is explained in more detail below.

### FA_gene algorithm: finding abnormal genes in autism

**Fig. 1:**
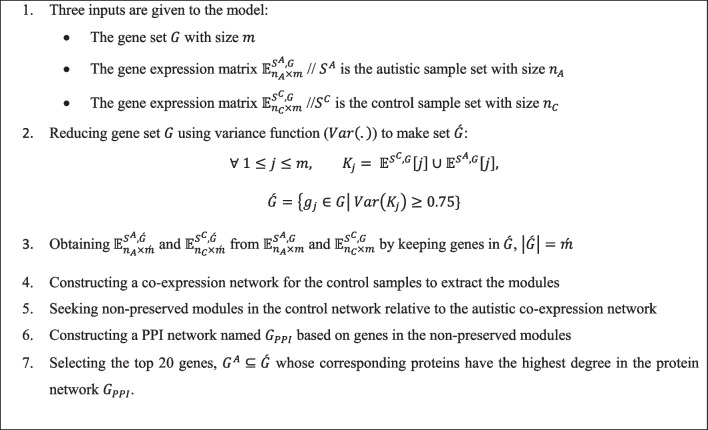
FA_gene algorithm to find important genes involved in autism

We propose an algorithm for finding a small set of genes, $G^{A} \subseteq G$, involved in autism named FA_gene (see Fig. 1). In the following, we illustrate this algorithm in more detail.

In the first step, the set $G$ with 18,801 genes and two gene expression matrices ${\mathbb{E}}_{21 \times 18801}^{{S^{A} {,}G}}$ and ${\mathbb{E}}_{21 \times 18801}^{{S^{C} {,}G}}$ are given as inputs to the FA_gene algorithm for finding genes involved in autism. The second step extracts a gene set $\mathop G\limits^{\prime } \subseteq G$ with 4704 genes, $\mathop m\limits^{\prime } = 4707$ where $\mathop m\limits^{\prime } \ll m$. Set $\mathop G\limits^{\prime }$ includes genes whose expressions are diverse between control and autistic samples. Then, the third step generates ${\mathbb{E}}_{{n_{A} \times \mathop m\limits^{\prime } }}^{{S^{A} ,\mathop G\limits^{\prime } }}$ and ${\mathbb{E}}_{{n_{C} \times \mathop m\limits^{\prime } }}^{{S^{C} ,\mathop G\limits^{\prime } }}$ based on ${\mathbb{E}}_{{n_{A} \times m}}^{{S^{A} ,G}}$ and ${\mathbb{E}}_{{n_{C} \times m}}^{{S^{C} ,G}}$ by keeping genes in $\mathop G\limits^{\prime }$ (See Additional file 1, Additional file 2).

In the fourth step, the algorithm uses WGCNA v. 1.70.3 package [25] to construct the control co-expression network as the reference network for ${\mathbb{E}}_{21 \times 4704}^{{S^{C} {,}\mathop G\limits^{\prime } }}$ matrix. In order to do this, the “adjacency” function is applied for building an adjacency matrix from the gene expression matrix according to the following parameters: type = “signed hybrid”, power = 8, corFnc = “bicor”. Then, the adjacency matrix is converted to the Topological Overlap Matrix (TOM) for achieving the dissimilarity matrix (1-TOM) by the “TOMsimilarity” function. At the end of this step, we use the “flashClust” and “cutreeDynamic” functions from the flashClust [26] package in R to extract modules from the reference network based on the dissimilarity matrix. Also, the “cutreeDynamic” function is performed according to “minModuleSize” = 30 and “deepSplit” = 2 parameters to cluster the tightly connected genes into groups called modules. We find 12 modules named using colors. Genes with no module are gathered in a group named “Gray”.

The fifth step seeks the modules with $Z_{Summery}$ value less than 2 as non-preserved modules, according to [27]. The $Z_{Summery}$ statistic is defined based on two other statistics, $Z_{density}$ and $Z_{connectivity}$. The $Z_{density}$ statistic shows whether nodes in a module in the reference network are connected as high as in the test network. The $Z_{connectivity}$ statistic determines the similarity of the connectivity pattern between nodes in each module of the reference and test networks. So, to find modules that are not reproducible (are non-preserved) from control to autism, we consider the control network as a reference and the autistic network as a test network. We calculate $Z_{Summery}$ statistic using “modulePreservation” function with parameters: type = “signed hybrid”, corFnc = “bicor”, nPermutations = 200. At the end of this step, the “Turquoise” module is found with 1173 genes as the non-preserved module.

In the sixth step, a PPI network named $G_{PPI}$ is constructed based on the genes in the “Turquoise” module by “STRINGdb” [28] package in R to find genes with the most critical roles in the module. This package finds 1021 proteins. So, a network is achieved with 1021 nodes and 18,286 edges. (For $G_{PPI}$, see Additional file 3).

In the last step, 20 genes whose corresponding proteins have the highest degree in the protein network $G_{PPI}$ are collected in the set $G^{A}$ as abnormal genes in autism.

### DMN_miRNA algorithm: detecting the minimum number of miRNAs in autism

**Fig. 2:**
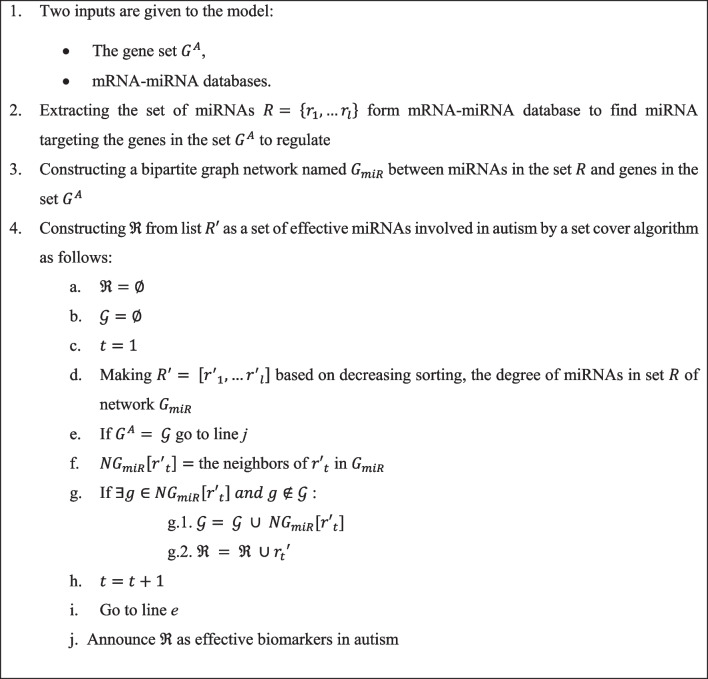
DMN_miRNA algorithm to detect the minimum number of miRNAs in autism

We propose the DMN_miRNA algorithm (see Fig. 2) for detecting the set of miRNAs $\mathop R\limits^{\prime } \subseteq R$ to target abnormal genes in the autism for regulation.

In the first step, the set $G^{A}$ as abnormal genes and three mRNA–miRNA databases, Tarbase v8.0 [29], mirTarbase v8.0 [30] and miRecords [31], are given as inputs. These databases contain mRNA and their miRNA regulators, whose regulatory relationships were validated experimentally or computationally. In the second step, we submit set $G^{A}$ to an online tool called miRNet [32] for retrieving mRNA–miRNA pairs from these databases. In this regard, the set $R = \left\{ {r_{1} , \ldots r_{l} } \right\}$, $l = 35$, is obtained for genes in the set $G^{A}$.

In the third step, we use the miRNet website to construct a bipartite network called $G_{miR}$. This network includes two node sets based on miRNA set $R$ and the abnormal gene set $G^{A}$. Each edge from the miRNA $r \in R$ to the gene $g \in G^{A}$ shows that $r$ targets $g$ to regulate. $G_{miR}$ is available in Additional file 4.

In the last step, we would like to obtain a minimum set of effective biomarkers (miRNAs), $\Re \subseteq R$, which regulate genes in the $G_{miR}$. In other words, we are looking for the smallest set of miRNAs whose gene targets cover $G^{A}$. So, we consider $G^{A}$ as the set that is desired to be covered, target genes of each miRNA as a subset and perform an extended greedy set cover algorithm on the network $G_{miR}$. According to this definition, every subset assigns to a miRNA. Therefore, we are selecting the smallest set of miRNAs that covers $G^{A}$ by finding the minimum set cover.

## Results

In this section, we show the selected miRNAs as regulators for abnormal genes in autism by performing the proposed framework.

### Finding abnormal genes involved in autism

We perform the FA_gene algorithm (see Method section) to find a small set of genes involved in autism. Based on the second step of the algorithm, we reduce 18,801 genes to 4707 $\left( {\mathop G\limits^{\prime } } \right)$ due to the variation in gene expression of control and autistic samples.

**Fig. 3:**
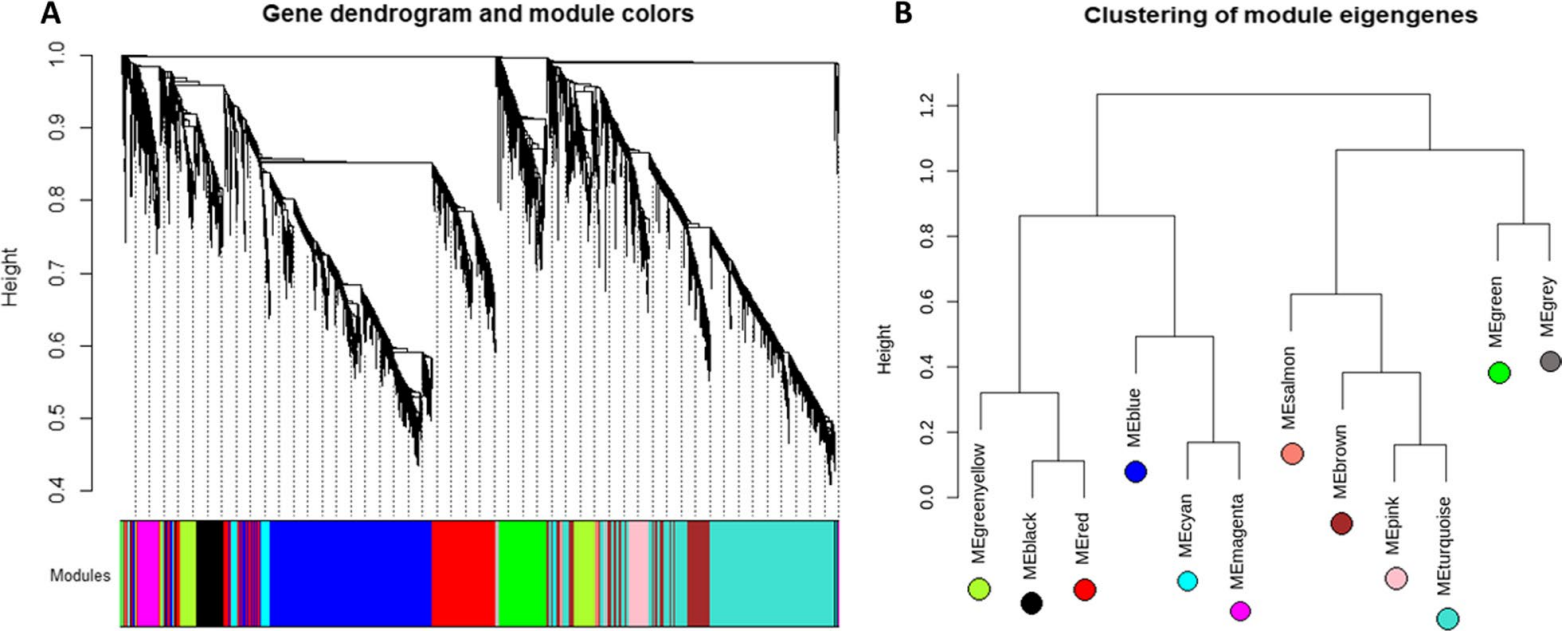
Hierarchical clustering dendrogram of genes in the control network (x-axis) based on dissimilarity between them (y-axis); each main branch forms a module, and the corresponding module is named by a color (**A**). Clustering of modules eigengenes (**B**)

According to the fourth step of the algorithm, we use set $\mathop G\limits^{\prime }$ to construct the co-expression control network as a reference network to extract modules. Figure 3A shows the 12 detected modules from the gene co-expression network. Figure 3B displays the inter-modular relationships between greenyellow, black and red modules, blue, cyan, and magenta modules, salmon, brown, pink, and turquoise modules, green and gray modules.

**Fig. 4:**
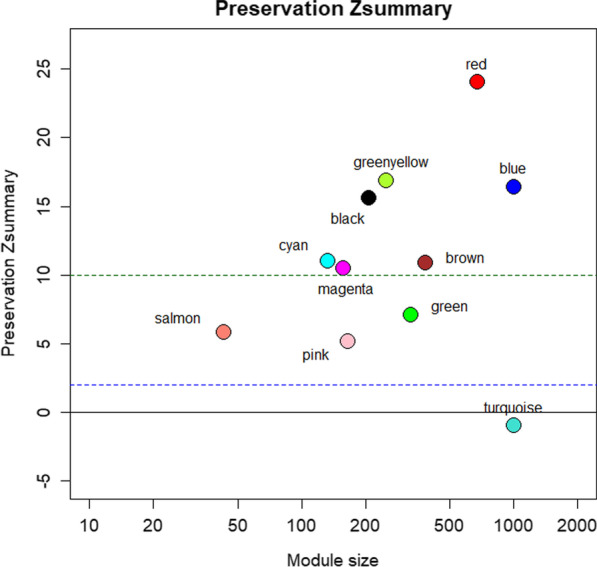
The module preservation based on ${\text{Z}}_{{{\text{Summery}}}}$ statistic (y-axis) and module size (x-axis)

Based on the fifth step of the FA_gene algorithm, we evaluate the preservation of modules between ASD and control samples by $$Z_{Summery}$$. Figure 4 shows the preservation $$Z_{Summery}$$ plot which includes the correlation between module size (genes) and $$Z_{Summery}$$. According to [27], modules with $$Z_{Summery}$$ less than 2 can be considered as non-preserved modules between reference (control) and test (autism) co-expression networks. As it can be seen in the figure, all modules are preserved except for the “Turquoise” module, with $$Z_{Summery} = - 0.94$$. This module is valuable because it is distinct in autism and control co-expression networks. This module in the control co-expression network includes $${\raise0.7ex\hbox{$1$} \!\mathord{\left/ {\vphantom {1 4}}\right.\kern-0pt} \!\lower0.7ex\hbox{$4$}}$$ number of genes in the control co-expression network (1173 out of 4704 nodes) and is also dense with 651,335 edges. While it has 1127 nodes and 64,566 edges in the autistic network. In comparison with the autistic network, 49 nodes and 586,769 connections are lost.

**Fig. 5:**
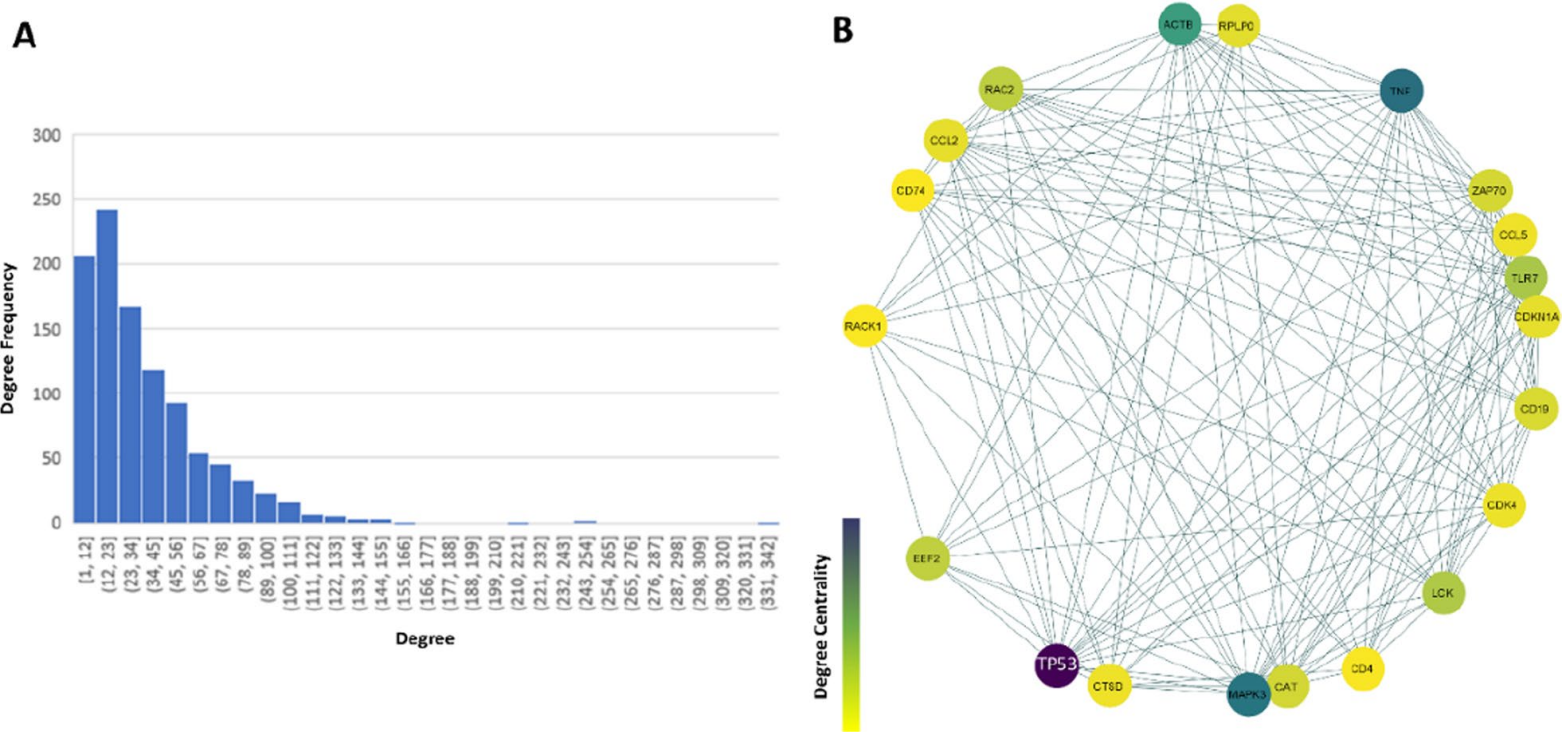
Degree histogram for nodes in ${\text{G}}_{{{\text{PPI}}}}$ (**A**), the extracted PPI sub-network ${\text{G}}_{{{\text{PPI}}}}$ based on the degree centralities (**B**)

According to the sixth step of the algorithm, we make the PPI network by the corresponding genes in the “Turquoise” module to find important genes whose abnormal expression may lead to the loss of the nodes and connections in the non-preserved co-expression module. A PPI network, $G_{PPI}$, with 1021 nodes (proteins) and 18,286 edges achieved for 1173 genes. Figure 5A shows the degree distribution of nodes in the PPI network. Most nodes (more than 600 nodes) have degrees less than 50. The maximum degree is 333, and the minimum degree is 1. Figure 5B displays the sub-network of $G_{PPI}$ on the subset of top 20 nodes with the highest degrees which is plotted using Cytoscape 3.9.1 [33]. The nodes in the extracted sub-network are colored based on the degree of centrality values. The set $G^{A}$ is obtained based on the top 20 corresponding proteins from $G_{PPI}$. Also, these top 20 nodes with the highest degree in $G_{PPI}$ with their corresponding degree are listed in Table 2.

**Table 2:** Genes in the set ${\text{G}}^{{\text{A}}}$ with the corresponding degree in ${\text{G}}_{{{\text{PPI}}}}$ and Turquoise module (${\text{TM}})$

Gene	$\user2{Deg}_{{\user2{G}_{{\user2{PPI}}} }}$	$\user2{Deg}_{{\user2{TM}}}$	Gene	$Deg_{{G_{PPI} }}$	$Deg_{TM}$
TP53	333	1150	CD19	134	890
TNF	253	1157	RPLP0	129	1161
MAPK3	248	1157	CDKN1A	127	1135
ACTB	213	1162	CCL2	127	979
TLR7	158	1087	CDK4	124	1162
LCK	155	1163	CCL5	123	1162
RAC2	148	1164	CTSD	122	1166
EEF2	148	1160	CD4	118	1030
CAT	137	1144	RACK1	117	1159
ZAP70	136	1147	CD74	117	1164

### Finding the minimum set of miRNAs to regulate abnormal genes in autism

We perform DMN_miRNA (see Method section) to find the minimum number of miRNAs targeting abnormal genes in autism for regulation. Based on the second step of the DMN_miRNA algorithm, set $R$ is constructed by 35 miRNAs, $\left| R \right| = 35$, which are retrieved from the miRNet website [32] as the regulator of genes in the set $G^{A}$. There is no miRNA regulator for LCK, CD19, and ZAP70 genes in the set $G^{A}$. So, the analysis is followed on 17 out of 20 genes in the set $G^{A}$.

**Fig. 6:**
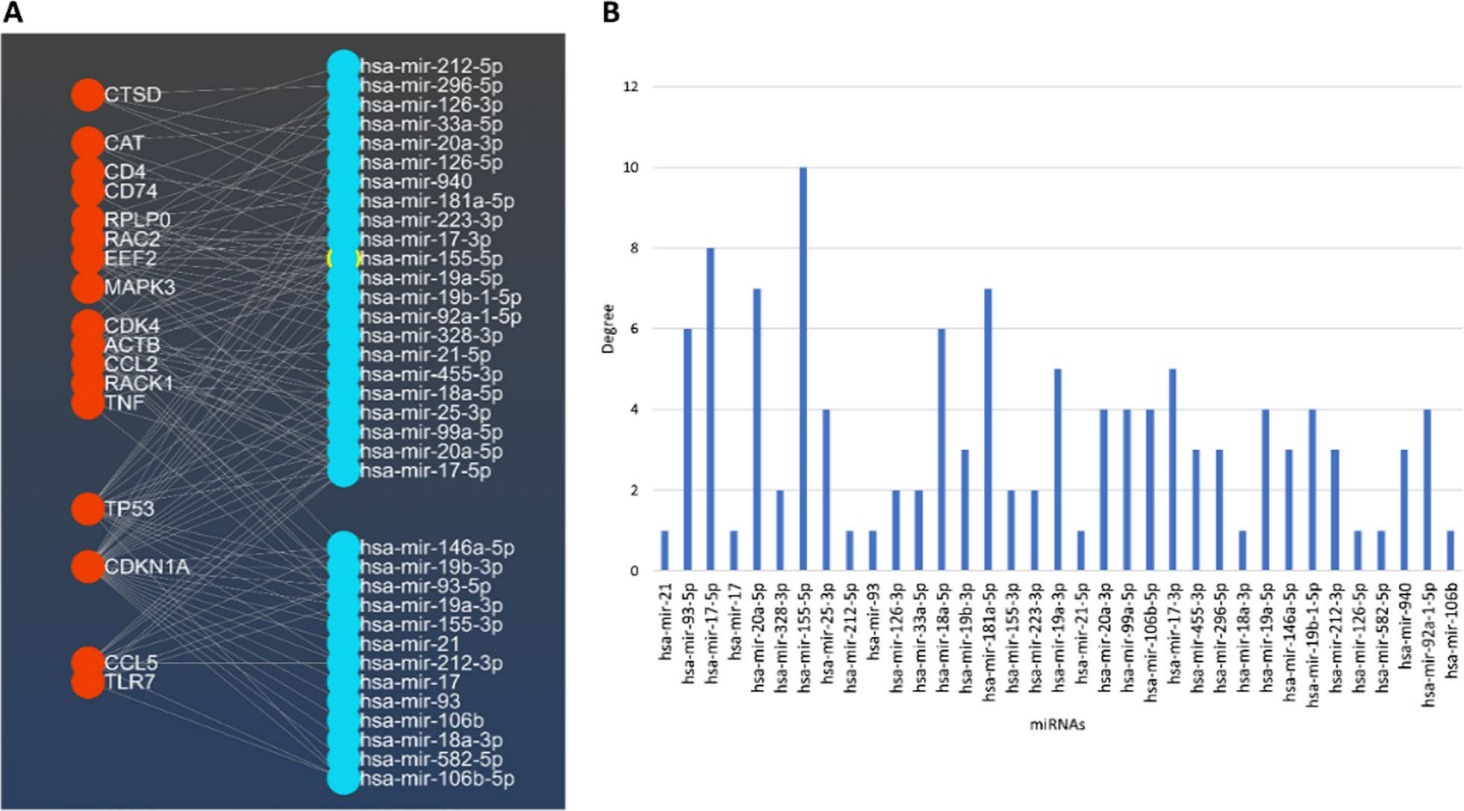
mRNA–miRNA network for genes in ${\text{G}}^{{\text{A}}}$; red circles represent genes, and blue ones represent miRNAs in ${\text{R}}$ (**A**). miRNAs in ${\text{R}}$ and the corresponding degree in ${\text{G}}_{{{\text{miR}}}}$ (**B**)

According to the third step of the algorithm, the bipartite network $G_{miR}$ is constructed with 17 genes, 35 miRNAs, and 119 edges (see Fig. 6A). The List of miRNAs in set $R$ and corresponding degrees in the network, $G_{miR}$, is available in Fig. 6B. Then, critical nodes based on the set cover algorithm (step fourth of the algorithm) are predicted as the effective biomarkers for autism and named $\Re = \{$hsa-mir-155-5p, hsa-mir-17-5p, hsa-mir-181a-5p, hsa-mir-18a-5p, hsa-mir-92a-1-5p$\}$. Table 3 shows the miRNAs in the set $\Re$, their degrees, and their target genes in $G_{miR}$.

**Table 3:** miRNAs in $\Re$; corresponding degree and targets in ${\text{G}}_{{{\text{miR}}}}$

miRNA	$Deg_{{G_{miR} }}$	Target genes
hsa-mir-155-5p	10	CDK4, ACTB, TNF, CAT, TP53, RAC2, RPLP0, CDKN1A, EEF2, CCL
hsa-mir-17-5p	8	MAPK3, TNF, TLR7, ACTB, CDK4, TP53, CCL5, CDKN1A
hsa-mir-181a-5p	7	MAPK3, ACTB, CTSD, TP53, RPLP0, CDKN1A, CD4
hsa-mir-18a-5p	6	CDK4, ACTB, TP53, RACK1, CDKN1A, EEF2
hsa-mir-92a-1-5p	4	CD74, TP53, CDKN1A, EEF2

We also submit miRNA set $\Re$ to miRNet to extract all target genes (7498 genes), which are regulated by set $\Re$. These target genes are used to evaluate the regulatory impact of identified miRNAs and for further analysis.

To investigate the influence of miRNA set $\Re$ in autism, a list of autism-related genes is collected based on the extracted genes from [34] and autism-related genes in the SFARI (Simons Foundation Autism Research Initiative) human genes database [35]. This list contains 1079 genes and is named *autistic genes*. By comparing the genes in this list with extracted targets from miRNet, we found that the miRNAs in the set $\Re$ regulate 822 from the *autistic genes*. Therefore, considering that a large number of *autistic genes* (about 76%) are affected by these miRNAs, they can play a significant role in autism.

### Enrichment analysis of target genes for predicted miRNAs

In this section, to ensure the effectiveness of the miRNAs (set $\Re$) in Biological Processes (BP) and pathways related to autism, we perform Gene Ontologies (GOs) and pathway analysis for all target genes (7498 genes), which are obtained using miRNet.

The online tool DAVID (Database for Annotation Visualization and Integrated Discovery) [36, 37] was used to extract biological processes and KEGG (Kyoto Encyclopedia of Genes and Genomes) pathways [38] for all gene targets of the set $\Re$. We select the top 10 BPs and pathways according to the repeated target genes to compare them with the BPs and pathways of *autistic genes*.

**Fig. 7:**
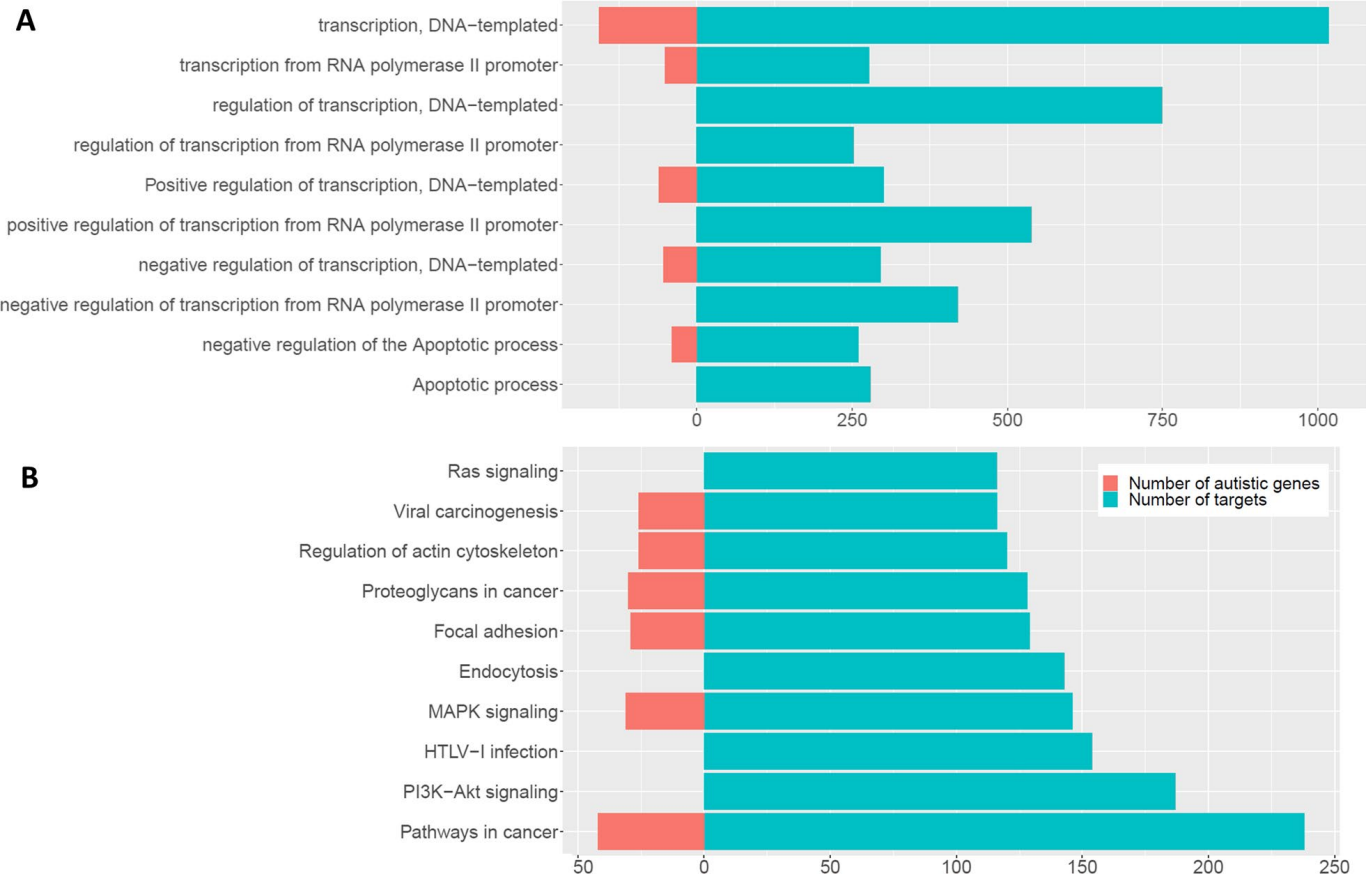
Enrichment analysis for miRNAs in R: Biological process (**A**), Pathway analysis (**B**)

The corresponding occurrences of target genes and *autistic genes* in each BP and pathway are shown in Fig. 7A and B, respectively. Focusing on the event of *autistic genes* indicates that there are five common BPs with gene targets of $\Re ,$ which are positive and negative regulation of transcription, DNA-templated, transcription from RNA polymerase II promoter, and negative regulation of the apoptotic process. Although three BPs are not observed in *autistic genes,* they are all about the regulation of transcription from RNA polymerase II promoters. Also, six of the top 10 pathways are common between *autistic genes* and gene targets: pathways in cancer, MAPK signaling, focal adhesion, regulation acting cytoskeleton, viral carcinogenesis, and proteoglycans in cancer.

## Discussion

In this study, we proposed a two-step framework including two algorithms (FA_gene and DMN_miRNA) to find critical genes and the minimum number of miRNAs involved in autism. The low number of samples and the high number of genes are always challenging in studying gene expression datasets. There are different ways to reduce the number of genes, mainly by using machine learning and statistical methods. However, there is a risk of overfitting for reducing the number of genes by machine learning methods. Meanwhile, most statistical tests do not consider the biological relationships between genes. Therefore, in the FA_gene algorithm, we made a co-expression network of control samples by considering positive correlation values between gene expressions to cluster genes into modules with the same expression pattern to find the relations between genes. Then, the non-preserved module, compared with the autistic network, was detected, which was the biggest module with 1173 genes. The PPI network of these genes was constructed to find critical genes in this module that encoded proteins with crucial roles inside the cells. A small number of highly connected proteins in the PPI network can maintain the global network structure and lead us to essential proteins [39] in the non-preserved module. The expression of the top 20 nodes ($G^{A}$) in this PPI network were compared between autism and control samples. Although, these genes were not in the list of DEGs but eleven of them (TP53, TNF, MAPK3, ACTB, RAC2, ZAP70, CD19, CDKN1A, CDK4, CTSD, CD74) were downregulated in the autism *vs* control samples with a logFC value between -0.36 to -0.8. On the other hand, we searched the literature to find the relationships between genes in $G^{A}$ and autism or any other neurological disorder. TP53 as the highest degree node was identified as a critical driver in ASD [3]. Also, developmental abnormalities were detected in altered TP53 and TP53-associated pathways in autism [40]. A high level of TP53 was detected in some brain areas in autistic samples *vs* controls [41]. TNF was related to autism [42], and TNF high blood concentration was observed in autistic children associated with symptom severity [43]. MAPK3 was associated with autism in several genome-wide association studies, copy number variants studies [44, 45], and also one of the biomarkers proposed to detect the early stage of Alzheimer [46]. ACTB is another gene related to autism [35]. Studies displayed that mutations in ACTB can cause Baraitser-Winter syndrome [47]. TLR7 was related to cognition and dendrite growth [48]. Studies showed that LCK is downregulated in the whole blood of ASD patients compared to controls [49]. RAC2 is a member of the Rho family GTPases. This family has an important role in brain development and is implicated in some neuropsychiatric and neurodegenerative diseases [50]. Also, its downregulation in Schizophrenia was reported [51]. Variations and differentially expression of EEF2 cause neurodevelopmental disorders like Alzheimer [52, 53]. CAT encodes catalase enzyme, which is decreased in autistic patients [54]. ZAP70 is another gene that is downregulated in the whole blood of ASD patients compared to controls [49]. CD19 is a biomarker for normal and neoplastic B cells, as well as follicular dendritic cells [55]. A statistically decrease in the number of B cells (CD19) in persons with Alzheimer's disease was depicted [56]. CDKN1A was identified as a critical driver in ASD [3, 57]. The higher levels of CCL2 and CCL5 were detected in ASD patients while lower plasma levels of these genes were detected in ASDs with fragile X syndrome [58–60]. CDK4 expression is decreased in the acute state of schizophrenia [61]. CTSD is one of 55 gene signatures for autism according to [9] and was reported related to autism [42]. CD4 protein is an important mediator of neuronal damages in central nervous system [62]. CD74 is another gene that inhibits β-Amyloid production [63], and β-Amyloid is amplified in autistic patients [64]. A decrease in RACK1 was reported in the cortex of down syndrome patients [65]. There is evidence that a decrease in the PKC activity in the aging brain or Alzheimer is related to the loss of RACK1 [66]. All the above reports demonstrate that genes in $G^{A}$ are related to autism, other neurological issues, or the nervous system. Although the association of these genes with autism is not entirely determined yet, they could be a potential signature for autism since all of them except for RPLP0 are reported to be involved in neurological issues.

In the second step of the proposed framework, we used the DMN_miRNA algorithm to make an mRNA–miRNA network constructed based on the selected genes and corresponding regulators. miRNAs are key post-transcriptional regulators in different cellular biological processes, which led us to evaluate common miRNAs as critical regulators in ASD. Instead of finding miRNAs as regulators for all genes in the non-preserved module, we focus on the genes in $G^{A}$. Since genes of $G^{A}$ are connected (positively correlated) to almost all nodes (1170 out of 1173 genes) in the module, it can be assumed that there are common miRNA regulators for genes in $G^{A}$ and the rest of the genes in the module. Thus, by investigating an mRNA–miRNA network reduced to genes in $G^{A}$ and their regulators, a minimum set of miRNAs can be obtained that target almost all genes in the non-preserved module. To find a small set (as much as possible) of miRNAs that covers all selected genes, we used a set cover algorithm, with a bit of change in the definition of the original set cover. Our algorithm found five miRNAs that were reported to be related to autism by previous studies. The upregulation of “mir-155-5p” was reported in the cerebellar cortex [67], amygdala [68], and frontal cortex [69]. The “miR-17” and “miR-181a” were up-regulated in peripheral blood [70] and lymphoblastoid cell lines [71], respectively. Also, the upregulation of “miR-181a” was reported in lymphoblastoid cell lines [71]. These reports were in agreement with our results, which showed that most genes in $G^{A}$ which are targeted by these miRNAs were downregulated in autism according to control samples. These miRNAs enrich pathways that are reported related to autism by previous studies. “Pathways in cancer” was introduced as one of the common pathways between autism and cancer [72]. The relation of “endocytosis” to autism was reported in [73]. The involvement of “focal adhesion” and “actin cytoskeleton” in the pathogenesis of autism was announced by [74]. Although relation of “Regulation acting cytoskeleton”, “Viral carcinogenesis,” and “Proteoglycans in cancer” were not found in the litrature but these pathways were common with *autistic-gene* pathways. Therefore, 9 out of 10 top pathways for the miRNA set $\Re$ were specially reported or potentially related to autism. It shows that miRNAs in $\Re$ should have a regulatory role in autism.

## Conclusions

In this study, using both co-expression and PPI networks led us to critical genes in ASD, which were reported as genes related to the nervous system or neurological disorders but couldn’t be detected with DEG analysis. This set of critical genes (TP53, TNF, MAPK3, ACTB, TLR7, LCK, RAC2, EEF2, CAT, ZAP70, CD19, RPLP0, CDKN1A, CCL2, CDK4, CCL5, CTSD, CD4, RACK1, CD74) were co-expressed with almost all genes in the non-preserved module of co-expression network that guided us to assume common miRNA regulators for most genes in this module. The DMN_miRNA algorithm detected a minimum number of (five) miRNA regulators for these critical genes; hsa-mir-155-5p, hsa-mir-17-5p, hsa-mir-181a-5p, hsa-mir-18a-5p, hsa-mir-92a-1-5p. These miRNAs regulate the most involved genes in autism which were approved by the previous studies and enrichment analyses. So, we suggest using FA_gene and DMN_miRNA algorithms to detect critical genes and a minimum number of miRNAs from expression analysis. The critical genes and regulators for ASDs indicated in this study can be examined in experimental studies and then suggested for diagnostic tests.

## Supplementary Information

**Additional file 1.** The autistic gene expression matrix (${\mathbb{E}}_{{n_{A} \times \mathop m\limits^{\prime } }}^{{S^{A} ,\mathop G\limits^{\prime } }}$).

**Additional file 2.** The control gene expression matrix (${\mathbb{E}}_{{n_{C} \times \mathop m\limits^{\prime } }}^{{S^{C} ,\mathop G\limits^{\prime } }}$).

**Additional file 3.** The PPI network ($G_{PPI}$) for genes in the non-preserved module.

**Additional file 4.** The mRNA–miRNA network ($G_{miR}$) for genes in $G^{A}$.

## Data Availability

The gene expression dataset analyzed in this study was extracted from Gene Expression Omnibus (GEO) and is available under Accession Number GSE18123.

## Abbreviations

ASD: Autism spectrum disorder
FA_gene: Finding abnormal genes in autism
DMN_miRNA: Detecting the minimum number of miRNAs in autism
GBPSO-SVM: Geometric binary particle swarm optimization-support vector machine
mRNA: Messenger RNAs
DEG: Differentially expressed gene
PPI: Protein–protein interaction
GEO: Gene expression omnibus
PDD-NOS: Pervasive developmental disorder-not otherwise specified
TOM: Topological overlap matrix
SFARI: Simons foundation autism research initiative
BP: Biological processes
GO: Gene ontologies
DAVID: Database for annotation visualization and integrated discovery
KEGG: Kyoto encyclopedia of genes and genomes
